# Reliability and Inter-Device Agreement Between a Portable Handheld Ultrasound Scanner and a Conventional Ultrasound System for Assessing the Thickness of the Rectus Femoris and Vastus Intermedius

**DOI:** 10.3390/jfmk10030299

**Published:** 2025-08-01

**Authors:** Carlante Emerson, Hyun K. Kim, Brian A. Irving, Efthymios Papadopoulos

**Affiliations:** School of Kinesiology, Louisiana State University, Baton Rouge, LA 70803, USA; cemers2@lsu.edu (C.E.); hkkim@lsu.edu (H.K.K.); brianairving@lsu.edu (B.A.I.)

**Keywords:** ultrasound, skeletal muscle, muscle hypertrophy, sarcopenia

## Abstract

**Background**: Ultrasound (U/S) can be used to evaluate skeletal muscle characteristics in clinical and sports settings. Handheld U/S devices have recently emerged as a cheaper and portable alternative to conventional U/S systems. However, further research is warranted on their reliability. We assessed the reliability and inter-device agreement between a handheld U/S device (Clarius L15 HD_3_) and a more conventional U/S system (GE LOGIQ *e*) for measuring the thickness of the rectus femoris (RF) and vastus intermedius (VI). **Methods**: Cross-sectional images of the RF and VI muscles were obtained in 20 participants by two assessors, and on two separate occasions by one of those assessors, using the Clarius L15 HD_3_ and GE LOGIQ *e* devices. RF and VI thickness measurements were obtained to determine the intra-rater reliability, inter-rater reliability, and inter-device agreement. **Results**: All intraclass correlation coefficients (ICCs) were above 0.9 for intra-rater reliability (range: 0.94 to 0.97), inter-rater reliability (ICC: 0.97), and inter-device agreement (ICC: 0.98) when comparing the two devices in assessing RF and VI thickness. For the RF, the Bland–Altman plot revealed a mean difference of 0.06 ± 0.07 cm, with limits of agreement ranging from 0.21 to −0.09, whereas for the VI, the Bland–Altman plot showed a mean difference of 0.07 ± 0.10 cm, with limits of agreement ranging from 0.27 to −0.13. **Conclusions**: The handheld Clarius L15 HD_3_ was reliable and demonstrated high agreement with the more conventional GE LOGIQ *e* for assessing the thickness of the RF and VI in young, healthy adults.

## 1. Introduction

Strategies for reliably and routinely assessing skeletal muscle characteristics are of relevance to clinical and sports settings. From a clinical perspective, the assessment of skeletal muscle mass combined with measures of physical function is used to identify individuals with sarcopenia [1]. Sarcopenia is an age-related syndrome associated with a higher risk of falls and fractures [2], hospitalizations [3], cognitive impairment [4], osteopenia [5], metabolic syndrome [6], and other adverse outcomes, including all-cause mortality [7]. From an athletic/sports perspective, the ability to accurately assess chronic skeletal muscle adaptations in response to training, as well as morphologic alterations associated with previous or ongoing skeletal muscle injuries, is important and can inform training and rehabilitation strategies to optimize performance.

Computed tomography (CT), magnetic resonance imaging (MRI), and dual X-ray absorptiometry (DXA) are well-established techniques for assessing musculoskeletal characteristics, particularly in clinical settings. However, they all exhibit major limitations, such as high costs and lack of portability [8,9]. Additionally, routine use of CT is limited by its ionizing radiation [8,9]. Therefore, the frequent use of CT, MRI, and/or DXA to assess and monitor changes in skeletal muscle characteristics exhibits several challenges.

Several studies suggest that ultrasonography may be used as an alternative and more practical strategy for researchers and clinicians than the conventional and cumbersome imaging techniques (e.g., CT, MRI and DXA) for assessing muscle mass of selected muscle groups as opposed to whole-body muscle mass [9,10,11]. Ultrasound (U/S) has widely been used to assess muscle quantity (mass) via muscle thickness of the lower extremities, particularly the rectus femoris (RF) and vastus intermedius (VI) across different populations [12,13,14]. Specifically, the RF and VI have been used to monitor adaptations in response to resistance training [14] predict postoperative outcomes [12] and confirm the presence of sarcopenia in adults [13]. Additionally, greater muscle thickness of the RF and VI has been associated with lower pain scores and better function among adults with knee osteoarthritis [15]. Conventional U/S systems may be costly and not portable. The Clarius L15 HD_3_ (Clarius Mobile Health, Vancouver, BC, Canada) is a relatively inexpensive, pocket-sized, high-frequency, wireless, handheld ultrasound scanner that has the potential to efficiently and reliably assess skeletal muscle parameters in clinical and sports settings. However, to our knowledge the reliability of the Clarius L15 HD_3_ and its agreement with a conventional U/S system for measuring the thickness of the RF and VI have not been previously examined.

Measures of muscle mass are often used along with measures of muscle strength to provide a more thorough assessment of physical fitness and performance in athletes and to confirm the presence of sarcopenia in older adults [16]. However, correlations between measures of muscle mass and muscle strength present conflicting findings across different populations. For example, meta-analytic data demonstrated weak correlations between lower limb muscle strength and lower limb muscle size measured by U/S, CT, or MRI (r = 0.26) in adults [17]. Similarly, weak correlations between muscle strength and measures of muscle mass using DXA or the D3-creatine dilution method have been observed in older adults (r = 0.19–0.32) [18]. However, strong correlations between the 1-RM and lean body mass (r > 0.75) have been found in adult powerlifters [19]. The disparate findings on the relationship between muscle strength and muscle mass among studies may relate to differences in population characteristics and the methods employed to assess muscle mass. Thus, further research is warranted to understand the relationship between muscle mass and strength.

The primary aim of this study was to assess the reliability and inter-device agreement of the Clarius L15 HD_3_ with a more conventional U/S system (GE LOGIQ *e*, GE Healthcare, Chicago, IL, USA) by comparing thickness measurements of the RF and VI. Our secondary aim was to examine correlations between lower body strength based on the one-repetition maximum (1-RM) for leg extension and ultrasound-based thickness of the RF and VI.

## 2. Materials and Methods

### 2.1. Participants

Participants were eligible if they were between 18 and 40 years old, capable and willing to provide informed consent, and had no medical conditions based on the Physical Activity Readiness Questionnaire for Everyone (PARQ+) [20]. All interested individuals provided written informed consent before any data collection. Eligible participants were also asked to provide information on their physical activity levels using the International Physical Activity Questionnaire—Short Form (IPAQ-SF) [21]. Using non-probability sampling, our aim was to recruit thirty (n = 30) participants, of whom the first ten (n = 10) would allow standardization of operator training between the two assessors despite differences in their level of prior experience with ultrasonography. Specifically, the first assessor was a graduate student with three years of clinical research experience but no prior formal U/S training, whereas the second assessor was a scientist and Certified Clinical Densitometrist with over 20 years of experience in performing and analyzing body composition data, and over 5 years of experience using U/S. Thus, data from twenty (n = 20) participants were used for the present analysis, a sample size that is in line with previous literature [9,10,22,23]. All study procedures were approved by the Institutional Review Board (IRBAM-23-1397).

### 2.2. Training for Standardization of Ultrasound Measurements

Practice sessions were conducted to standardize U/S measurements prior to formal data collection and to familiarize the inexperienced operator with image acquisition using the U/S scanners, in line with previous work [22]. Specifically, the experienced assessor (BI) provided an orientation session that introduced the inexperienced assessor (CE) to the interface of the Clarius L15 HD_3_ (Clarius Mobile Health, Vancouver, BC, Canada) and GE LOGIQ *e* (GE Healthcare, Chicago, IL, USA), identification of the anatomical landmark for placement of the probe, probe positioning, and common issues related to image acquisition and identification of the skeletal muscles of interest. Subsequently, both assessors performed measurements on 10 subjects. During image acquisition of those 10 subjects, the experienced assessor (BI) had the opportunity to supervise the inexperienced assessor (CE) and discuss any technical discrepancies that would undermine standardization of image acquisition (e.g., position of the limb, pressure of the probe, amount of gel used, and anatomical landmarks for measurements). Following completion of the 10 subjects, assessors performed U/S measurements on 20 subjects independently. Overall, the inexperienced assessor had approximately 10 h of training prior to formal data collection.

### 2.3. Study Procedures

The study involved two visits to the Exercise Physiology Laboratory within the School of Kinesiology at Louisiana State University, separated by 5–7 days. All visits were conducted between 8:00 am and 2:00 pm and each participant visited the lab at the same time for both visits. Participants were instructed to avoid engaging in exercise for ≤12 h before each visit while keeping the amount of time since last engaging in exercise as consistent as possible for both visits.

During the first visit, participants’ anthropometric characteristics were measured using standard techniques. Before initiation of all U/S assessments, participants were required to rest while lying in the supine position for at least 15 min to avoid fluid shifts. During this rest period, landmarks were established for U/S location. Specifically, using Gulick tape, the distance between the greater trochanter and the lateral condyle of the right femur was measured and markers were placed at the midpoint so that ultrasonography could be performed at the anatomical region of interest [10]. Two assessors independently performed femur length measurement and midpoint identification (i.e., after one assessor completed his U/S assessment, the femur midpoint landmarks were removed so that the second assessor could establish his own landmarks). After 15 min of rest, participants’ RF and VI thickness of the right leg was independently assessed by the two assessors using both the Clarius L15 HD3 and GE LOGIQ *e* 12 L linear probes. Each assessor independently quantified participants’ RF and VI thickness using the built-in software of each U/S unit deriving measurements in real time. The assessors were not allowed to be present in the room together during study visits, including when measurements were taken. Specifically, the research coordinator would notify the second assessor to enter the room after completion of measurements by the other assessor. The data obtained from each assessor were extracted from the study coordinator and stored until analysis. Following the U/S measurements of the first visit, participants underwent 1-RM testing of leg extension of the right leg using standard procedures in line with previous work [24]. Specifically, participants performed a warm-up set of 8–10 repetitions at 40–50% of their perceived 1-RM followed. After 2 min of rest, participants were asked to perform another set of 5–6 repetitions at 60–70% of their perceived 1-RM. Following another 2 min rest, a third set of 3 repetitions at ~80% of the participants’ perceived maximum was introduced. Subsequently, 3 to 4 sets of a single repetition were used to determine the participant’s true 1-RM.

Approximately 5–7 days after the first visit, participants returned to the Exercise Physiology Laboratory for their second and final visit. During the second visit, the U/S measurements of the right RF and VI using both the Clarius L15 HD_3_ and GE LOGIQ *e* were repeated only by one assessor.

### 2.4. Ultrasound Assessment and Muscle Thickness Measurement

The same technique was utilized for the Clarius L15 HD_3_ and GE LOGIQ *e* U/S units to capture three cross-sectional images displaying the RF, VI, and femur. A generous amount of ultrasound gel was applied at the anatomical location of interest. With the probe or device oriented in the correct direction (i.e., perpendicular to the long axis of the thigh with correct mediolateral orientation), minimal pressure was applied to capture an image where the RF, VI, and femur were as closely in line as possible without being compressed. Specifically, the skin should have a slightly convex shape rather than appear as a straight line, which is indicative of minimal compression. Once a desirable image was identified, the image was frozen and stored if it appeared satisfactory upon further scrutiny. After at least three satisfactory images were captured with one ultrasound unit, the assessor repeated the same process with the other unit. In some cases, it was necessary to manually manipulate the orientation of the participant’s leg (i.e., internally or externally rotate the femur) to capture the desired image. In these instances, efforts were made to keep the leg orientation for a given participant consistent for both visits.

Both ultrasound units were utilized in the Brightness Mode (B-mode) setting with Gain 45 dB, and depth 5 cm. However, the depth was adjusted when necessary, based on the thickness of a participant’s subcutaneous adipose tissue and muscle to allow visibility of the femur. Given the interindividual differences among study participants, the depth ranged from 5 to 7 cm. Notably, the maximal depth of the Clarius L15 HD_3_ unit is 7 cm.

**Figure 1:**
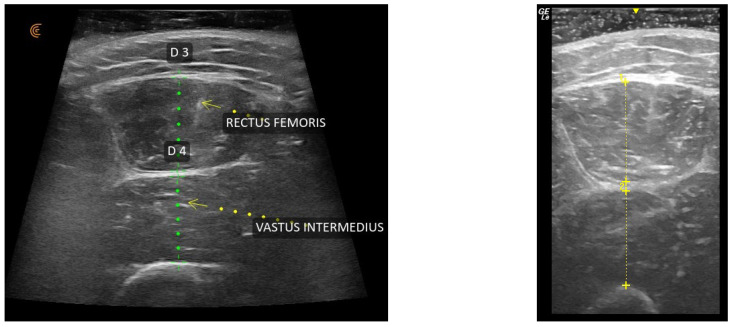
Thickness of the rectus femoris and vastus intermedius using the Clarius L15 HD_3_ (**left image**) and GE LOGIQ *e* (**right image**).

Using the built-in software of the two U/S scanners, the thickest aspects of the RF and VI were measured independently (Figure 1). Specifically, the thickness of the RF and VI were measured between the superficial and deep aponeuroses [10]. The three measurements were completed on three separate images from each of the two ultrasound units. The average of the three measurements per muscle group obtained independently by the two assessors using each of the U/S scanners were used for the analysis.

### 2.5. Statistical Analysis

The characteristics of study participants at baseline were summarized using the mean and standard deviation for continuous data, whereas frequencies and proportions were used for categorical data. Normality of the data was assessed using the Kolmogorov–Smirnov test. Intra-rater (first and second visit measurements obtained by the first assessor) and inter-rater reliability (first visit measurements between assessors) for evaluating the repeatability of RF and VI muscle thickness measurements using the Clarius L15 HD_3_ and GE LOGIQ *e* was assessed using the intraclass correlation coefficient (ICC_3,1_) based on a two-way mixed-effects model with absolute agreement. The percentage differences were calculated by calculating the percentage differences between two raters (100 × (measurement 1 − measurement 2) / measurement 1). Typical error (TE, cm) was calculated as the standard deviation (SD) of the difference divided by the square root of 2 [9]. The standard error of measurement (SEM) was calculated by multiplying the SD of the differences between measurements by the square root of one minus the ICC and then multiplying by 100. Additionally, we calculated the minimal detectable change (MDC) by multiplying the SEM by the square root of two and then by 1.96. Potential differences in the measurements between the two examiners were assessed using independent samples *t*-test. The Bland–Altman method was employed to assess the agreement between the two ultrasound devices (Clarius L15 HD_3_ and GE LOGIQ *e*) for each muscle using the average values obtained by the first assessor at both study visits. Pearson’s correlation coefficient (*r*) was utilized to measure the strength and direction of the relationship between muscle thickness and 1-RM measures. Additionally, least significant change (LSC) scores were calculated to determine the minimum change that would be considered ‘real’ (beyond measurement error) at the 95% confidence level consistent with the International Society for Clinical Densitometry’s Precision Assessment for each U/S device [25]. Specifically, we used the ISCD Advanced Precision Calculating Tool (https://iscd.org/learn/resources/calculators/, accessed on 10 June 2024) to calculate the LSC scores for each U/S device using the data collected from by the same sonographer on the two separate study visits (V1 and V2). LSC scores are presented as LSC for the root-mean-square standard deviation (RMS_SD_) and root-mean-square percent coefficient of variation (RMS_%CV_) at 95% confidence, LSC_RMSD_ (2.77*RMS_SD_) and LSC_%CV_ (2.77*RMS_%CV_) respectively, as recommended by the International Society of Clinical Densitometry [25,26]. The present LSC scores were calculated from twenty participants with duplicate measurements, while the ISCD recommends 30 participants with duplicate measurements for calculating LSC scores for clinical bone densitometry [25]. Moreover, the present LSC scores were calculated based on duplicate measurements on different study visits, including additional between-day variability. All data were analyzed by a member of the study team (HKK) who was not present during image acquisition and processing. All analyses were performed using IBM SPSS version 28.0 (IBM Corp., Armonk, NY, USA).

## 3. Results

Of the 34 participants who signed informed consent, one was ineligible due to a chronic condition based on the PARQ+, while three participants lost to follow-up prior to their first visit. Twenty (n = 20) participants (mean age: 21.9 years) were included in the analysis, of whom sixteen were females (80%). On average, participants engaged in 48 min of moderate intensity and 187 minutes of vigorous intensity physical activity per week. The characteristics of study participants at baseline are listed in Table 1.

### 3.1. Intra-Rater Reliability

Table 2 lists the results of the intra-rater reliability for assessing thickness of the RF and VI via the Clarius L15 HD_3_ and GE LOGIQ *e*. The intra-rater reliability was assessed only for the assessor who was present in both study visits for all study participants. All ICCs for intra-rater reliability in assessing RF and VI muscle thickness were above 0.9, ranging from 0.94 to 0.97, indicating excellent test–retest reliability for both U/S scanners [27]. Percentage differences in RF and VI muscle thickness ranged from −4.16% to 1.08%, generally showing slightly lower values in the first visit compared to the second. TE (cm) ranged from 0.09 to 0.12, with the highest TE observed for RF thickness using the GE LOGIQ e.

### 3.2. Inter-Rater Reliability

Table 3 lists the results of the inter-rater reliability for assessing thickness of the RF and VI at the first visit using the Clarius L15 HD_3_ and GE LOGIQ *e*. All ICCs for inter-rater reliability were above 0.95, which is suggestive of excellent inter-rater reliability [27]. Percentage differences in RF and VI muscle thickness using the Clarius L15 HD_3_ were 1.43% and −1.33%, respectively. Similarly, percentage differences in RF and VI muscle thickness using the GE LOGIQ *e* were 2.86 and −0.30%, respectively. TE (cm) ranged from 0.09 to 0.11, with the largest TE observed for VI thickness using the Clarius L15 HD_3._

### 3.3. Inter-Device Agreement

**Figure 2:**
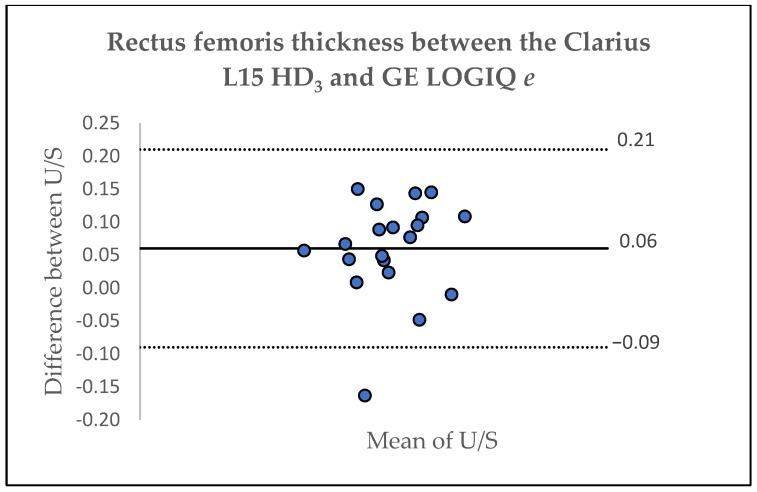
Bland–Altman Plot depicting the agreement for the evaluation of rectus femoris thickness for the Clarius L15 HD_3_ and GE LOGIQ *e*.

**Figure 3:**
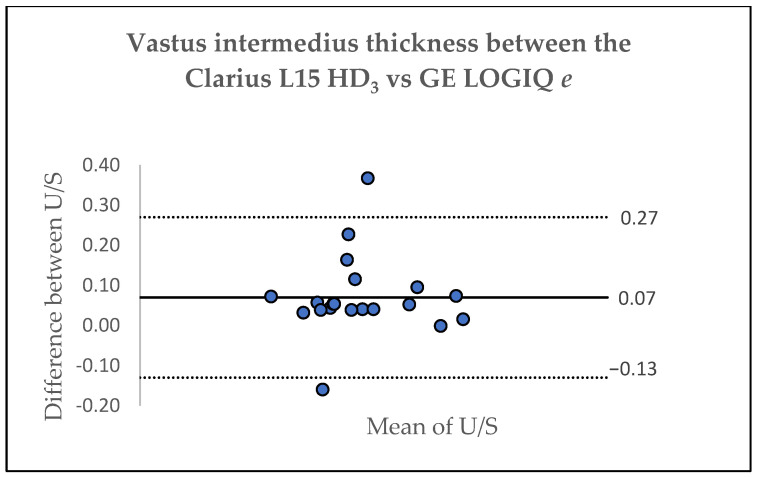
Bland–Altman Plot depicting the agreement for the evaluation of vastus intermedius thickness for the Clarius L15 HD_3_ and GE LOGIQ *e*.

Table 4 lists the results of the inter-device agreement between the Clarius L15 HD_3_ and the GE LOGIQ *e* for assessing thickness of the RF and VI based on one assessor. The ICC for RF and VI was 0.98 (95%CI: 0.94–0.99) and 0.98 (95%CI: 0.95–0.99), respectively, indicating excellent agreement between the two scanners [28]. A Bland–Altman analysis was also used to assess the agreement between the Clarius L15 HD_3_ and the GE LOGIQ *e*, showing acceptable levels of agreement. For the RF, the Bland–Altman plot revealed a mean difference of 0.06 ± 0.07 cm, with limits of agreement ranging from 0.21 to −0.09, whereas for the VI, the Bland–Altman plot showed a mean difference of 0.07 ± 0.10 cm, with limits of agreement ranging from 0.27 to −0.13 (Figure 2 and Figure 3). However, as shown in Figure 3, two data points were outside the limits of agreement.

### 3.4. Precision

Table 2 also lists the precision analysis results for assessing the RF and VI thickness via the Clarius L15 HD_3_ and GE LOGIQ *e*. The GE LOGIQ *e* had higher RMS_SD_ and RMS_%CV_ than the Clarius L15 HD_3_ for the between-visit replicates of the RF and VI thicknesses. Subsequently, the GE LOGIQ *e* had higher LSC_RMSD_ and LSC_%CV_ than the Clarius L15 HD_3_ for the between-visit replicates of the RF and VI thicknesses.

### 3.5. Correlations Between Muscle Thickness and 1-RM for Leg Extension

**Figure 4:**
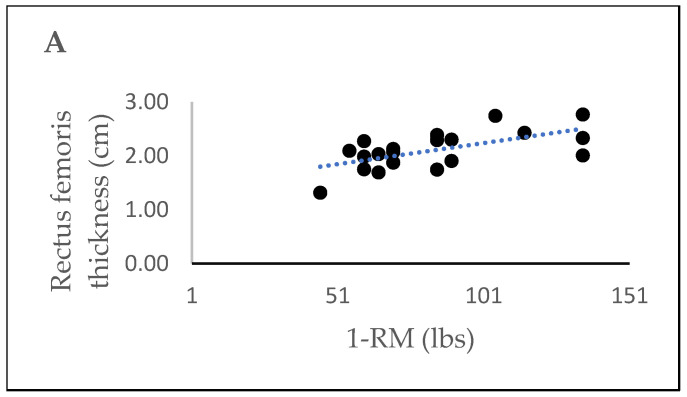

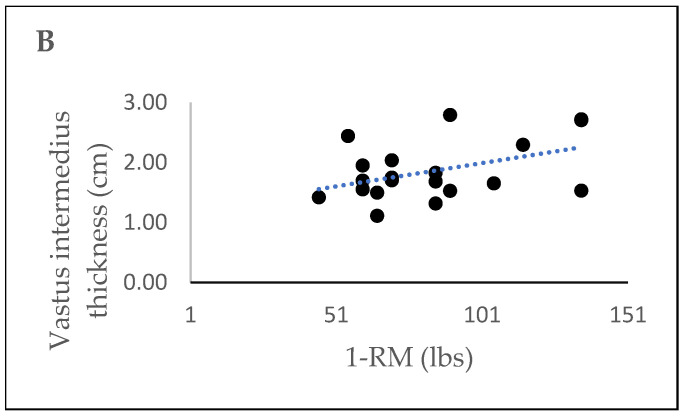
(**A**,**B**) Relationship between the 1-RM and thickness of the rectus femoris (**A**) and the vastus intermedius (**B**).

Moderate significant correlations were found between the 1-RM of leg extension and RF thickness (*r* = 0.620, *p* = 0.004), while weaker correlations were demonstrated between the 1-RM and VI thickness (*r* = 0.442, *p* = 0.051) (Figure 4A,B). Additionally, a partial correlation was conducted to examine the relationship between muscle thickness and 1-RM, while controlling for sex. A statistically significant partial correlation was found between RF thickness and 1-RM (*r* = 0.558, *p* = 0.013). However, after accounting for the effect of sex, VI thickness showed a weak correlation with 1-RM (*r* = 0.290, *p* = 0.229).

## 4. Discussion

This study assessed the reliability and inter-device agreement between a portable, pocket-sized handheld U/S scanner (Clarius L15 HD_3_) and a conventional U/S (GE LOGIQ *e*) for assessing the thickness of the RF and VI. Our findings suggest that Clarius L15 HD_3_ can accurately and reliably measure thickness of the lower extremities.

We found excellent test–retest reliability of both U/S systems for estimating thickness of the RF and VI. Specifically, the ICC using the Clarius L15 HD_3_ ranged from 0.96 to 0.97. Previous work using a different handheld, portable U/S device corroborates our findings, suggesting that handheld U/S can reliably measure muscle thickness of the RF in healthy adults [29,30]. Similarly, portable U/S may be used to assess muscle thickness in older adults, given the age-associated declines in muscle mass and function. For example, in study of 150 community-dwelling older adults, a portable U/S system using A-mode demonstrated good intra-rater reliability for thickness of the triceps, biceps, anterior thigh and calf muscles with ICCs ranging from 0.80 to 0.90 [31]. Similarly, the intra-rater reliability using the GE LOGIQ *e* was also excellent, ranging from 0.94 to 0.97.

The inter-rater reliability for measuring the thickness of the RF and VI was excellent using the Clarius L15 HD_3_ or the GE LOGIQ *e* (ICC: 0.97), despite the different level of experience between the two examiners with U/S image acquisition and analyses. To attenuate some of these differences, the first examiner who was less experienced with U/S imaging compared to the second examiner, was able to practice and improve his technique using the first ten subjects. Moreover, the second examiner also had the opportunity to familiarize himself with acquiring images using the new hand-held U/S device. Previous work has demonstrated that the level of experience with U/S image acquisition and analyses can influence U/S measures. Specifically, Carr and colleagues found good-to-excellent inter-rater reliability for image acquisition of muscle size using panoramic imaging, but poor reliability for analysis of muscle thickness [22]. According to the authors, the poor inter-rater reliability might relate to inconsistencies with the identification of muscle boundaries by the novice examiner who had less than two hours of instruction before image analyses [22]. In line with these findings, poor inter-rater reliability was found in another study where two novice examiners measured the thickness of the RF and VI in healthy adult males [29]. However, Oliveira and colleagues found moderate-to-good inter-rater reliability (ICCs: 0.70–0.76) of a handheld, portable U/S for assessing thickness of muscle groups of the upper and lower extremities in older adults [31]. Our findings highlight the importance of practice for novice examiners prior to U/S image acquisition and analysis that appears to optimize inter-rater reliability.

Furthermore, the inter-device agreement between the Clarius L15 HD_3_ and the GE LOGIQ *e* was also excellent for measuring thickness of the RF and VI (ICC: 0.98 for both the RF and VI). Nonetheless, measurements of the VI for two participants were outside the limits of agreement which may relate to positioning of the limb or the probe which could have affected the measurements. This underscores the importance of standardization protocols to minimize such occurrences in future studies. Our findings correspond with those by Turton and colleagues who also demonstrated inter-device agreement between a different handheld U/S device and a more conventional U/S system for measuring thickness of the RF and VI in healthy males [32]. Nonetheless, the level of agreement between the examined U/S scanners was lower in that study compared to our findings [32]. Similarly, a handheld U/S scanner exhibited high inter-device agreement with a conventional U/S system for measuring thickness of the RF (ICC: 0.82) and the VI (ICC: 0.89) in active healthy participants [30].

The ISCD has been a long-time proponent of using precision analysis to assess the test–retest reliability of dual X-Ray absorptiometry bone mineral density measurements in clinical practice at the level of the technologist [25,33]. More recently, investigators have begun reporting precision analysis for DXA-derived body composition measurements including lean body mass. A review identified six studies that reported LSC_RMSD_ and LSC_%CV_ for lean body mass in non-athletes and athletes using DXA [34]. The LSC_%CV_ ranged from 0.8% to 4.5% with 95% confidence [34]. Our results suggest that LSC%_CV_ is between 12.5 and 18.3% with 95% confidence for measuring RF and VI muscle thicknesses with the Clarius L15 HD_3_ and the GE LOGIQ *e* U/S systems. The higher LSC_%CV_ observed in the present study using U/S to measure muscle thickness of the RF and VI relative to the LSC_%CV_ obtained by DXA for measuring lean body mass are likely to be due to a few factors. First, small differences in DXA measurements of lean body mass led to a small RMS_%CV_ and thus a small LSC_%CV_ due to the relatively high lean body mass (i.e., large denominator), while small differences in skeletal muscle thickness led to a high RMS_%CV_ and thus a high LSC_%CV_ due to the relatively small muscle thickness (i.e., small denominator). Second, most of the aforementioned DXA studies performed same-day precision analysis, while the present study used non-consecutive-day (5–7 days apart) precision analysis, likely increasing variability. Consistent with this notion, one study that examined the precision of DXA-measured leg lean mass showed a ~37% increase in LSC_%CV_ when replicates were measured on consecutive days compared to when measured on the same day [35]. Few U/S studies have reported precision analysis for measuring muscle thickness, particularly with respect to reporting either LSC_RMSD_ or LSC_%CV_. However, a recent study reported raw %CV for measurements of the anterior thigh muscle (i.e., RF) in older adults with type 2 diabetes, older adult matched controls, and healthy young adults [36]. The raw %CV ranged from 0.4 to 1.3 for repeated measurements with their expert sonographer, compared to our 4.5 to 6.6% for RMS_%CV_ for our trained but novice sonographer. As noted above, our higher RMS_%CV_ is likely due to measurements taken on non-consecutive days. Moreover, it is unclear whether the repeated measurements conducted in the prior study repositioned their participants before taking repeated measurements as recommended by the ISCD [36]. In the absence of repositioning, raw %CV and/or RMS_%CV_ are likely to be considerably lower than with repositioning. Finally, additional training and the inclusion of more study participants (e.g., 30 participants with duplicate measurements) would likely lower the LSC_RMSD_ and LSC_%CV_.

Correlations between 1-RM leg extension and measures of muscle thickness were moderate in line with previous work [37,38], indicating the importance of assessing muscle strength in addition to muscle thickness. The moderate correlation between RF thickness and leg extension 1-RM may be attributed to neural factors such as differences in motor unit recruitment, rate of activation, synchronization, in addition to coordination of antagonistic muscle groups. Another possible mechanism relates to potential differences in fat infiltration of the skeletal muscle (i.e., myosteatosis), which has been shown to negatively affect muscle strength [39]. This may be particularly useful for the evaluation of sarcopenia according to the European Working Group of Sarcopenia in Older People 2, which defines sarcopenia as the combination of low muscle strength in addition to low muscle quantity or quality [16].

Our findings strengthen the rationale for using handheld, portable U/S devices for assessing muscle thickness in younger and older adults. Emerging evidence supports the use of handheld, portable U/S scanners for detecting sarcopenia [40] or assisting with the diagnosis of musculoskeletal injuries such as rotator cuff tears [41] and carpal tunnel syndrome [42], carotid artery plaque [43] and ulcerative colitis [44].

Our study includes several limitations. First, our results should be interpreted with caution due to the small sample size which may affect our study outcomes [45]. In particular, the study sample (n = 20) does not meet the sample size recommendations for calculating the LSC per the ISCD (n = 30) [25]. Thus, the precision and generalizability of our results regarding the LSC should be interpreted cautiously given that statistical power was insufficient. However, our sample size is in line with several studies that examined the reliability and inter-device agreement of U/S scanners for assessing skeletal muscle characteristics [9,10,22,23]. Second, the demographic characteristics of the participants (i.e., young healthy adults) limit the generalizability of our findings to other populations, particularly insufficiently active individuals or older adults, where conditions that impact skeletal muscle thickness such as sarcopenia and frailty are more relevant. Study assessors performed measurements independently, in real time following study visits to resemble a real-world scenario. A more rigorous strategy would involve randomization of images prior to obtaining measurements independently by each assessor. Additionally, the inclusion of additional U/S measures, such as cross-sectional area and echo intensity, would have provided further insight into the utility of the Clarius L15 HD_3_ in evaluating comprehensively the characteristics of the RF and VI.

## 5. Conclusions

A handheld portable U/S scanner can accurately and reliably assess the thickness of the RF and VI. Additional U/S precision studies are needed to further clarify whether U/S measurements can be used to detect clinically significant changes in skeletal muscle thickness and will likely help investigators deploy this cost-efficient method in multi-center clinical trials and/or epidemiological studies. Further studies are warranted to examine the role of handheld U/S devices in determining cross-sectional area and echo intensity that will provide further information on muscle quantity and quality, respectively.

## Figures and Tables

**Table 1:** Characteristics of study participants at baseline.

Characteristic	N = 20
Age, years (mean, SD)	21.9 (2.7)
Sex (females), n (%)	16 (80%)
Race, n (%)	
White	12 (60%)
Black	4 (20%)
Hispanic	2 (10%)
Asian	1 (10%)
Body mass index (kg/m^2^), mean (SD)	23.7 (4.2)
Days of vigorous PA per week (mean, SD)	2.8 (1.9)
Minutes of vigorous PA per week (mean, SD)	187 (167.3)
Days of moderate PA per week (mean, SD)	2.6 (1.8)
Minutes of moderate PA per week (mean, SD)	48 (31.0)
Days of walking per week (mean, SD)	5.9 (1.4)
Minutes of walking per week (mean, SD)	71.8 (77.9)
Minutes of sitting per week (mean, SD)	361.8 (142.9)

PA = Physical activity; SD = Standard deviation.

**Table 2:** Intra-rater reliability for measuring thickness of the rectus femoris and vastus intermedius using the Clarius L15 HD_3_ and GE LOGIQ *e*.

Thickness (cm)	Visit 1 (Mean, SD)	Visit 2 (Mean, SD)	Δ (%)	ICC (95%CI)	TE (cm)	RMS_SD_ (cm)	LSC_RMSD_ (cm)	RMS_%CV_	LSC_%CV_	SEM (%)	MDC (cm)
**RF** Clarius L15 HD_3_	2.11 (0.35)	2.13 (0.32)	−1.54 (6.59)	0.96 (0.90–0.98)	0.09	0.093	0.258	4.51	12.49	2.66	0.07
**RF** GE LOGIQ *e*	2.14 (0.38)	2.21 (0.33)	−3.91 (9.41)	0.94 (0.84–0.98)	0.12	0.122	0.339	6.23	17.26	4.02	0.11
**VI** Clarius L15 HD_3_	1.86 (0.49)	1.86 (0.41)	1.08 (9.44)	0.97 (0.92–0.99)	0.12	0.112	0.311	6.16	17.05	2.83	0.08
**VI** GE LOGIQ *e*	1.90 (0.45)	1.96 (0.45)	−4.16 (9.56)	0.97 (0.91–0.99)	0.11	0.116	0.321	6.61	18.31	2.65	0.07

ICC = intraclass correlation coefficient; MDC = minimal detectable change; RF = rectus femoris; SEM = standard error of measurement; TE = typical error; VI = vastus intermedius; RMS_SD_ = root mean square standard deviation; LSC_RMSD_ = least significant change for RMS_SD_ at 95% confidence; RMS_%CV_ = root mean square percentage coefficient of variation; LSC_%CV_ = least significant change for RMS_%CV_ at 95% confidence.

**Table 3:** Inter-rater reliability for measuring thickness of the rectus femoris and vastus intermedius using the Clarius L15 HD_3_ and GE LOGIQ *e*.

Thickness (cm)	Assessor 1 (Mean, SD)	Assessor 2 (Mean, SD)	Δ (%)	*p*	ICC (95%CI)	TE (cm)	SEM (%)	MDC (cm)
**Clarius L15 HD_3_**								
RF	2.11 (0.35)	2.08 (0.39)	1.43 (6.00)	0.38	0.97 (0.93–0.99)	0.09	2.20	0.06
VI	1.86 (0.49)	1.88 (0.46)	−1.33 (8.62)	0.68	0.97 (0.93–0.99)	0.11	2.72	0.08
**GE LOGIQ *e***								
RF	2.14 (0.38)	2.08 (0.39)	2.86 (7.02)	0.043	0.97 (0.91–0.99)	0.09	2.17	0.06
VI	1.90 (0.45)	1.90 (0.46)	−0.30 (10.27)	0.99	0.97 (0.93–0.99)	0.11	2.66	0.07

ICC = intraclass correlation coefficient; MDC = minimal detectable change; RF = rectus femoris; SEM = standard error of measurement; TE = typical error; VI = vastus intermedius.

**Table 4:** Inter-device agreement between the Clarius L15 HD_3_ and GE LOGIQ *e* for measuring thickness of the rectus femoris and vastus intermedius.

Thickness (cm)	Clarius L15 HD_3_ (Mean, SD)	GE LOGIQ *e* (Mean, SD)	Δ (%)	ICC (95%CI)	TE (cm)	SEM (%)	MDC (cm)
RF	2.11 (0.35)	2.14 (0.38)	−1.47 (6.20)	0.98 (0.94–0.99)	0.08	1.58	0.04
VI	1.86 (0.49)	1.90 (0.45)	−2.03 (7.20)	0.98 (0.95–0.99)	0.09	1.75	0.05

ICC = intraclass correlation coefficient; MDC = minimal detectable change; RF = rectus femoris; SEM = standard error of measurement; TE = typical error; VI = vastus intermedius.

## Data Availability

The data of this study are available from the corresponding author upon reasonable request.

## Abbreviations

The following abbreviations are used in this manuscript:

CT	Computed tomography
MRI	Magnetic resonance imaging
DXA	Dual X-Ray absorptiometry
U/S	Ultrasound
RF	Rectus femoris
VI	Vastus intermedius
PARQ+	Physical activity readiness questionnaire for everyone
IPAQ-SF	International Physical Activity Questionnaire-Short Form
1-RM	1-repetition-maximum
ICC	Intraclass correlation coefficient
TE	Typical error
SD	Standard deviation
SEM	Standard error of measurement
MDC	Minimal detectable change
LSC	Least significant change
ISCD	International Society for Clinical Densitometry
RMS_SD_	Root-mean-square standard deviation
RMS_%CV_	Root-mean-square coefficient of variation
